# Heterogeneity in the trajectories of psychological distress among late adolescents during the COVID‐19 pandemic

**DOI:** 10.1002/jcv2.12195

**Published:** 2023-08-24

**Authors:** Jean‐Philippe Gouin, Alejandro de la Torre‐Luque, Yolanda Sánchez‐Carro, Marie‐Claude Geoffroy, Cecilia Essau

**Affiliations:** ^1^ Department of Psychology Concordia University Montreal Quebec Canada; ^2^ Department of Legal Medicine, Psychiatry and Pathology Universidad Complutense de Madrid, CIBERSAM ISCIII Madrid Spain; ^3^ Center for Biomedical Research in Mental Health (CIBERSAM) Carlos III Health Institute Madrid Spain; ^4^ Department of Psychiatry McGill University and Douglas Mental Health University Institute Montreal Quebec Canada; ^5^ School of Psychology Roehampton University London UK

**Keywords:** adolescence, anxiety, COVID‐19, depression, loneliness, psychological distress, social support

## Abstract

**Background:**

The coronavirus disease 2019 (COVID‐19) pandemic has constrained opportunities in social, educational and professional domains, leading to developmental challenges for adolescents initiating their transition to adulthood. Meta‐analysis indicated that there was a small increase in psychological distress during the first year of the COVID‐19 pandemic. However, significant heterogeneity in the psychological response to the COVID‐19 pandemic was noted. Developmental antecedents as well as social processes may account for such heterogeneity. The goal of this study was to characterize trajectories of psychological distress in late adolescence during the COVID‐19 pandemic.

**Methods:**

5014 late adolescents born between 2000 and 2002 from the UK Millennium Cohort Study completed online self‐reported assessments at three occasions during the first year of the COVID‐19 pandemic (May 2020, September/October 2020 and February/March 2021). These surveys assessed psychological distress, loneliness, social support, family conflict, as well as other pandemic stressors. Information on developmental antecedents were obtained when cohort members were 17 years of age.

**Results:**

Four distinct trajectories class were identified. *Normative class* (52.13%) experienced low and decreasing levels of psychological distress, while *moderately increasing class* (31.84%) experienced a small, but significant increase in distress over time and *increasing class* (8.75%) exhibited a larger increase in distress after the first wave of the pandemic. *Inverted U‐shaped class* (7.29%) experienced elevated psychological distress during the first wave of the pandemic, followed by a decrease in distress in subsequent waves of the pandemic. Larger longitudinal increases in loneliness were noted among individuals in the elevated distress trajectory, compared to other trajectories. Pre‐pandemic psychopathology was associated with elevated distress early in the pandemic.

**Conclusions:**

The largest trajectory showed low and declining psychological distress, highlighting the resilience of the majority of late adolescents. However, a subgroup of adolescents experienced large increases in psychological distress, identifying a group of individuals more vulnerable to pandemic‐related stress.

## Graphical abstract

Trajectories of psychological distress during the COVID‐19 pandemic.

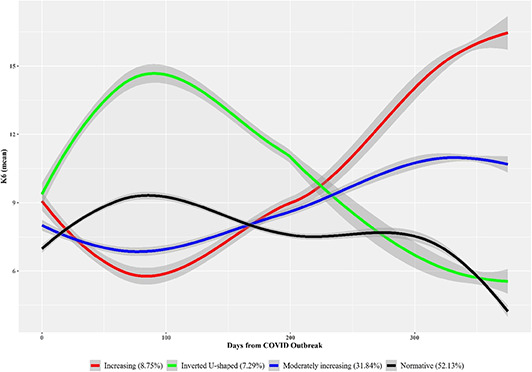

**Key points**

- Prior work with adolescents highlights significant heterogeneity in change in psychological distress during the COVID‐19 pandemic.
- Among late adolescents, increases in distress were small for about 30% of participants and large for about 9% of participants.
- About 8% of participants experienced elevated distress during the first wave followed by a subsequent decrease in distress.
- Individuals who experienced larger increase in psychological distress also reported concomitant increases in loneliness during the pandemic.
- Longitudinal follow‐ups are required to characterize the evolution of psychological distress once pandemic stressors abate.

## BACKGROUND

The public health and social measures (PHSMs) imposed during the coronavirus disease 2019 (COVID‐19) pandemic led to protracted disruptions in daily occupational, educational, and social activities, such as limited access to social and leisure activities, online schooling, unstable labor markets with recurrent closure of non‐essential businesses, and loss of career and educational opportunities (Lee et al., 2021; Sahu, 2020). Late adolescents and emerging adults (i.e., those aged 18–24 years) who are undergoing the transition to adulthood are negotiating novel developmental tasks including living independently, establishing their first romantic and sexual relationships, making educational and vocational choices, and beginning a working career (Arnett & Sugimura, 2014; Scales et al., 2016; Zarrett & Eccles, 2006). Pandemic‐related confinement measures may have constrained opportunities in social, romantic, sexual, educational, and vocational domains, creating unique challenges for late adolescents negotiating these various developmental tasks (Scales et al., 2016).

Several studies highlight that late adolescents reported higher levels of psychological distress during the first wave of the pandemic, compared to other age groups (Wetherall et al., 2022; World Health Organization, 2022). Specifically, it has been observed that late adolescents have experienced more depressive and anxiety symptoms than adults (30–59 years) and older adults (>60 years) (Sun et al., 2023; Wetherall et al., 2022). In addition, within this age group, an increase in psychological distress has been observed before and during the pandemic, particularly among girls (Madigan et al., 2023). Before the COVID‐19 pandemic it was estimated that 12.9% (Lu, 2019) and 11.6% (Tiirikainen et al., 2019) of young people had clinically significant symptoms of depression and anxiety, while during the pandemic these were rates of 25.2% and 20.4%, respectively (Racine et al., 2021). Similarly, in a longitudinal study of late adolescents with pre‐pandemic assessment in 2018, both depressive and anxious symptoms increased during the first 12 months of the COVID‐19 pandemic (Gouin et al., 2023). This contrasts with the typical decrease in psychological distress that have occurred among this age group in pre‐pandemic cohorts (Hangrove et al., 2020).

Meta‐analyses of longitudinal studies including pre‐pandemic data indicate small, but significant increases in psychological distress during the first few months of the pandemic, followed by a decline in distress toward the end of the first wave in the Spring 2020 (Kunzler et al., 2021; Madigan et al., 2023; Prati & Mancini, 2021; Robinson et al., 2022). Although most countries maintained some PHSMs during subsequent waves of the pandemic (Aknin et al., 2022), research examining the trajectories of psychological distress throughout the pandemic is limited. In longitudinal studies within the general population, fluctuations in psychological distress were observed during the first 15 months of the pandemic, largely in line with the waxing and waning indices of stringency of PHSMs and pandemic intensity (Aknin et al., 2022; Daly & Robinson, 2022; Ori et al., 2022). Several longitudinal studies of late adolescents reported a worsening of anxiety and depressive symptoms during the first 12 months of the COVID‐19 pandemic (Benatov et al., 2022; Hawke et al., 2021; Hu & Gutman, 2021; Patel et al., 2022; Rogowska et al., 2021; Stroud & Gutman, 2021). However, not all studies have observed protracted elevations in psychological distress among adolescents during this timeframe (Graupensperger et al., 2022; Rimfeld et al., 2021), highlighting the heterogeneity in psychological distress responses during the COVID‐19 pandemic.

Understanding variability in the psychological responses to the COVID‐19 pandemic and the related PHSM is important given the high risk for future pandemics (Marani et al., 2021). Characterizing trajectories of psychological distress and identifying their correlates will help us identify vulnerable individuals and potential mitigations strategies. Several risk and protective factors may explain differences in psychological distress during the COVID‐19 pandemic. In cross‐sectional and longitudinal studies, female adolescents reported on average more anxiety and depression than males (Del‐Valle et al., 2022; Stroud & Gutman, 2021). Ethnic and racial minority experienced more psychological distress than individuals from the majority group (Smith et al., 2020). Furthermore, financial stress exacerbated by the pandemic, that is, the worries about having enough money to meet their household's basic needs, have been associated with higher depression and anxiety among late adolescents over time (Ellwardt & Präg, 2021; Schoon & Henseke, 2022). Moreover, past studies indicate that pre‐pandemic mental health is a moderator of psychological distress during the pandemic. Individuals with severe anxiety and depressive symptoms pre‐pandemic displayed patterns of stable or decreasing symptoms during the first wave of the pandemic, whereas their counterparts with better pre‐pandemic mental health displayed patterns of increasing symptoms during the first wave of the pandemic (Bouter et al., 2022; De France et al., 2022; Hamza et al., 2021; Watkins‐Martin et al., 2021).

The quality of social relationships is also an important determinant of psychological distress across the lifespan. Late adolescence is usually associated with the expansion of social networks (Arnett & Sugimura, 2014; Miething et al., 2016). Pandemic‐related restrictions in in‐person interactions may have impacted distinct social processes that modulate risk for psychological distress (Foulkes & Blakemore, 2021). Loneliness is defined by the subjective and distressing perception of a discrepancy between the actual and desired quantity and quality of one's social relationships (Hawkley & Cacioppo, 2010). A meta‐analysis indicated that PHSMs were associated with increases in loneliness (Knox et al., 2022). Notably, late adolescents reported more loneliness than other age groups (Bu et al., 2020; Hu & Gutman, 2021; O’Connor et al., 2021; Varga et al., 2021). Outside of a pandemic context, loneliness is associated with elevated psychological distress (Beutel et al., 2017). Loneliness was also one of the strongest predictors of anxiety and depression during the first wave of the COVID‐19 pandemic (González‐Sanguino et al., 2020), including among late adolescents (Loades et al., 2020). Loneliness may thus be a key social process increasing risk for psychological distress among adolescents during the COVID‐19 pandemic.

Social support, the perception of the availability of close others to provide assistance in times of needs (Wethington & Kessler, 1986), is another social process that may modulate pandemic‐related psychological distress. Although loneliness and social support are conceptually related, empirical studies indicate that size of the correlation between the two constructs is small‐to‐moderate, indicating the unique contribution of each of these two social processes (Grey et al., 2020). Social support is an important protective factor against the development and worsening of mental health symptoms across the lifespan (Gariépy et al., 2016; Rueger et al., 2016), and especially during periods of transition or stress such as late adolescence (Pettit et al., 2011; Scardera et al., 2020). Social support may play a key role in supporting adaptive coping behaviors in response to various pandemic‐related stressors (Thoits, 1986). Higher social support was cross‐sectionally associated with decreased psychological distress during the COVID‐19 (Szkody et al., 2021). More social support was associated with faster decreases in psychological distress during the first wave of the pandemic (Amendola et al., 2021; Fluharty et al., 2021; Zhou et al., 2020), as well as lower depressive and anxiety symptoms, during the first year of the pandemic (Laham et al., 2021; Li et al., 2021). Within the general population, some studies suggest that social support is more stable than loneliness in the context of changing PHSMs (Laham et al., 2021; Xu et al., 2020), while other studies indicate that social support have increased during the pandemic (Luchetti et al., 2020). Given that the transition to adulthood is a key period for the expansion of social network, late adolescents may experience greater difficulties building or maintaining close social relationships than other age groups during periods of pandemic‐related restrictions in social activities, leading to cumulative negative impact on adolescents' mental health over time.

Finally, interpersonal stress may contribute to aggravate psychological distress under stressful conditions (Hammen, 2016). In particular, studies have provided some evidence of increased family conflict during lockdowns (Guo et al., 2020; Morgül, Kallitsoglou, Essau, Castro‐kemp, & Mateo, 2022). These family conflict, resulting from a wide range of stressors affecting family members, such as work from home/teleworking, loss of income, home‐schooling, and movement restrictions for young people to meet up with their friends, could increase risk for adolescents' psychological distress.

Taken together, prior studies indicate that, on average, a small increase in psychological distress was observed among late adolescents during the COVID‐19. However, significant heterogeneity in the psychological response to the pandemic were noted. Given key pivotal changes in social networks during this developmental period, changes in social support, loneliness, and family conflict may be key social processes underlying changes in distress among late adolescents during the COVID‐19 pandemic. Within the Millennium Cohort Study, significant increases in psychological distress were noted 12 months into the COVID‐19 pandemic, compared to the pre‐pandemic period (Patel et al., 2022). However, no study has examined heterogeneity in the change in psychological distress over time. Capitalizing on this longitudinal cohort design provides a unique opportunity to examine how previous psychopathology and current social functioning influences changes in psychological distress during the pandemic. The goals of the current study were thus to (a) examine the heterogeneity of change in psychological distress among late adolescents at 3 time points during the COVID‐19 pandemic, (b) examine change in social support and loneliness during the pandemic, and (c) to examine the contributions of pre‐pandemic psychopathology and other COVID‐19 and PHSMs‐related stressors to changes in psychological distress during the pandemic.

## METHOD

### Sample and data in analysis

Data from the Millennium Cohort Study (MCS) (Connelly & Platt, 2014) were used in this study. The MCS is a nationally‐representative birth cohort study aiming at depicting the developmental course of physical and mental health outcomes of British people born between 2000 and 2002 (i.e., the so‐called Generation Z). In terms of sampling, a stratified clustering strategy was followed to ensure adequate representation of ethnic minorities at the baseline assessment (i.e. 82% of participants were White, 2.5% were Indian, 4.8% were Pakistani, 2% were Bangladeshi, 1.3% were Black Caribbean, 2% were Black African, and 3% of cohort members had mixed ethnicity) (MCS; Connelly & Platt, 2014). The MCS comprises seven sweeps following cohort members from 9 months of age to age 17. In addition, data during the COVID‐19 pandemic were collected at three occasions (May 2020, September/October 2020 and February/March 2021). The first COVID‐19 survey took place in May 2020, representing 38 days of home confinement with universities, entertainment venues, hotels and any type of indoor leisure facilities had been closed by the UK government. The second COVID‐19 survey was conducted between September‐October 2020 while restaurants, pubs and other entertainment venues had been open since July 4, but opening hours were restricted and there were still social distancing measures such as the “rule of six” (The Health Foundation, 2023). The third assessment was between February‐March 2021. A new home confinement of the population had been decreed January 6^th^ but lifted on March 8^th^. Citizens were encouraged to stay in their locality. Outdoor recreation in public spaces between two people and outdoor gatherings of six people or two households were permitted (The Health Foundation, 2023). Further details on the COVID‐19 surveys can be consulted on the MCS webpage (https://cls.ucl.ac.uk/covid‐19‐survey/). All MCS protocols were approved by an ethical committee for human research (Shepherd & Gilbert, 2019).

The sample included in this study comprised 5014 late adolescents aged 19–21 years (60.59% female, mean age = 19.27, SD = 0.46) who responded to the psychological distress scale (i.e., the K6 scale below) at least once across the COVID‐19 survey waves. In terms of COVID‐19 survey response rate, 44.22% of the study sample responded to the first COVID‐19 survey; 56.42% of participants responded to the second survey and the response rate to the third survey was 81.27%.

### Measures

Sociodemographic (i.e., sex at birth, age, and ethnic group), pre‐COVID‐19 psychopathology and COVID‐19‐related (proximal factors) data were used in this study.

#### Demographic variables

Demographic variables assessed for all participants were sex at birth, age and ethnic group (subjects were classified as: white Caucasian or non‐white Caucasian) and country (i.e. England, Wales, Scotland and Ireland).

#### Psychopathology

Previous psychopathology factors were taken from the MCS 2018 sweep when cohort members were 17 years old. Specifically, the Strength and Difficulties Questionnaire (SDQ), self‐reported version (SDQ; Goodman et al., 1998) assessed internalizing and externalizing symptoms (i.e., the Difficulties scores) as well as prosocial behaviors. The SDQ is a 25‐item scale covering psychopathology symptoms from four dimensions (i.e., emotional symptoms, conduct problems, hyperactivity/inattention, and peer relationship problems) and prosocial behaviors. Each item can be responded using a 3‐point Likert scale. The SDQ showed acceptable levels of reliability in adolescents in a study previously conducted in five European countries (Cronbach's *α* = 0.71 for the Total Difficulties scale; and Cronbach's *α* = 0.71 for the Prosocial Behaviors scale) (Essau et al., 2012).

Moreover, self‐harm behavior at age 17 was considered. Self‐harm was measured by a single item (item: whether the cohort member had self‐hurt in the last year at least in one of these ways: bruising or pinching; burning, cutting or stabbing, taking an overdose of tablets, pulling out hair).

#### COVID‐related factors

Most of the COVID‐19‐related factors studied were measured by single items: whether the participant was infected by the SARS‐CoV‐2 virus during the study period (yes/no), whether the participant experienced more family conflict during the pandemic, compared to the pre‐pandemic, whether the participant experienced more financial difficulties during the first national lockdown (first COVID‐19 survey wave) in comparison to the pre‐pandemic period.

Furthermore, two time‐variant social processes were considered. First, perceived loneliness was measured across the three COVID‐19 surveys with the 3‐item University of California, Los Angeles (UCLA) Loneliness Scale (Russell, 1996). Items were rated on a 3‐point Likert scale. The 3‐item Social Provisions Scale (SPS) (Cutrona & Russell, 1983) measured the availability of social support across the COVID‐19 surveys. Reliability indexes of the 3‐item SPS in our sample ranged from Cronbach's *α* between 0.65 and 0.79 across the survey waves.

Finally, psychological distress across the COVID‐19 surveys was measured by the Kessler Distress Scale‐6 (Kessler et al., 2003). The K6 is made up of six items on a 5‐point Likert scale assessing anxiety and depressive symptoms in the past 30 days. The psychometric properties of the K6 were adequate in previous studies with adolescent and young adults populations (*α* = 0.84; Mewton et al., 2016).

### Data analysis

The overall course of psychological distress and time‐variant factors (i.e., loneliness and social support) was evaluated using repeated‐measure analysis of variance. To prevent inflated type I error due to large sample size (Lin et al., 2013), only differences with at least a medium effect size (I.E., *η* ^2^ ≥ 0.06) would be considered to be meaningful differences.

The heterogeneous trajectories of psychological distress throughout the first COVID‐19 pandemic year were identified using growth mixture modeling (GMM) (Proust‐lima & Liquet, 2017; Ram & Grimm, 2009)^.^ The K6 score across the MCS COVID‐19 surveys was used as an observed variable capturing the psychological distress latent process. Growth mixture modeling, as a person‐centered approach, allows relaxing of the assumption of a unitary course of development. Subject‐specific variability may be well captured by clustering individuals with a similar trajectory into the same group (class). For parameter estimation, robust maximum likelihood and full information methods were used, enabling the estimation of individual‐specific trajectories even when intermittent missing data were present. Days from the WHO's pandemic declaration (11 March 2022) was used to estimate the linear and quadratic time effects. Unconstrained GMM solutions were estimated to decrease the probability of class overestimation due to covariates (Hu et al., 2017; Vermunt, 2010). Growth mixture modeling solutions with an increasing number of trajectory classes were compared. The model with an optimal class enumeration was selected according to the following criteria: low sample‐adjusted Bayesian information criterion (SABIC) and Akaike information criterion (AIC), mean of posterior probabilities to belong to each identified class higher than 0.70; and meaningful proportion of participants within each class (5%) (Nylund‐Gibson et al., 2023; Spiegelhalter et al., 2002).

Multinomial logistic regression was used to study the associations between distress trajectory membership and sociodemographic (i.e., sex at birth and ethnic group), pre‐COVID‐19 psychopathology factors (i.e., SDQ total difficulties, SDQ prosocial score and self‐harm on the MCS 2018 sweep) and COVID‐related cross‐sectional predictors (i.e., SARS‐CoV‐2 virus infection, family conflict level, and financial difficulties in comparison to the pre‐pandemic period), entered simultaneously in the model. Finally, multilevel linear regression was used to study the relationship between distress trajectory class membership and the course of loneliness and social support during the first year of the pandemic, controlling for the above‐mentioned covariates. Note that small correlations were observed between the loneliness and social support scores across COVID‐19 survey waves (*r* = −0.23 in the first wave, *r* = −0.25 in the second wave and *r* = −0.17 in the third wave). The repeated measure factor was used as a multilevel factor. Linear and quadratic effects of time, as well as sociodemographic, pre‐COVID‐19 psychopathology, trajectory class membership factors and cross‐sectional predictors COVID‐19‐related factors were included in the model. In addition, time*trajectory class interaction effects were tested.

Regarding model fit for both multinomial logistic and multilevel linear regression, the AIC was estimated to assess whether the model with covariates fitted better than an unconstrained model. The relative risk ratio (*RRR*) for multinomial logistic regression and the *B* coefficient, for the multilevel linear regression, were used as covariate loading estimates. All analyses were conducted using R x64 3.0.1 (lcmm, mice, lmer4 and psych packages) and STATA 15.

## RESULTS

The Table 1 displays the descriptive statistics of sample. In total, a sample of 5014 participants was analyzed. Most participants were born in England (65.61%) and were White Caucasian (87.47%). Adolescent psychopathology at age 17 (MCS 2018 sweep X̄ = 11.31, sd = 5.56) was over the mean levels observed in other normative samples (i.e. German sample: X̄ = 10.93, sd = 4.9, Cypus sample: X̄ = 9.87, sd = 3.8, UK sample: X̄ = 10.87, sd = 2.9¸ Sweden sample: X̄ = 8.99, sd = 2.3 and Italy sample: X̄ = 10.26, sd = 2.3) (Essau et al., 2012). Moreover, more than one in four cohort members engaged in self‐harm behavior at age 17. Regarding psychological distress, there was a slightly increasing trend in K6 scores across the COVID‐19 survey waves, with a small effect size, F (1.97, 2537.12) = 16.47, *p* < 0.01, *η* ^2^ = 0.003. A slightly increasing levels of loneliness across waves was also observed, with a small effect size, F (1.94, 2546.98) = 18.25, *p* < 0.01, *η* ^2^ = 0.004. The repeated‐measure analysis of variance revealed the absence of change over time in perceived social support across the COVID‐19 survey waves, F (2, 2478) = 0.01, *p* = 0.99, *η* ^2^ < 0.001.

**TABLE 1:** Sociodemographic and psychopathological features of sample.

Variable	Statistic
Sex (%male)	39.41
Age at first COVID‐19 sweep	19.27 (0.46)
Ethnic group (%non‐white)	12.53
Country	
England	65.61
Wales	13.82
Scotland	11.88
Ireland	8.7
Pre‐COVID (mid‐adolescence) psychopathology	
Total difficulties^a^	11.31 (5.56)
Prosocial behavior^a^	8.04 (1.67)
Self‐harm at age 17 (%yes)	25.62
COVID‐19 factors	
SARS‐Cov‐2 infection (%yes)	12.93
Family conflict (%more conflict during the pandemic)	5.92
Financial management (%worse off management during the pandemic)	25.99
Psychological distress^b^	
COVID‐19 wave 1	8.40 (5.12)
COVID‐19 wave 2	8.44 (5.37)
COVID‐19 wave 3	8.61 (5.58)
Perceived loneliness^c^	
COVID‐19 wave 1	5.16 (1.70)
COVID‐19 wave 2	5.21 (1.77)
COVID‐19 wave 3	5.28 (1.80)
Social support^d^	
COVID‐19 wave 1	6.79 (0.65)
COVID‐19 wave 2	6.79 (0.68)
COVID‐19 wave 3	6.80 (0.70)

*Note*: Percentage of cases are displayed for dichotomous and categorical variables. Mean and standard deviation (between brackets) are displayed for continuous variables. The pre‐COVID data were collected in 2018, when cohort members were 17 years old. The COVID‐19 data were collected across three waves: COVID‐19 wave 1 (May 2020), COVID‐19 wave 2 (September/October 2020) and COVID‐19 wave 3 (February/March 2021).

^a^ Derived from the SDQ.

^b^ Coming from the Kessler K6 Scale (K6).

^c^ Derived from the 3‐item UCLA Loneliness Scale.

^d^ Derived from the 3‐item Social Provisions Scale.

### Trajectories of psychological distress

**FIGURE 1:**
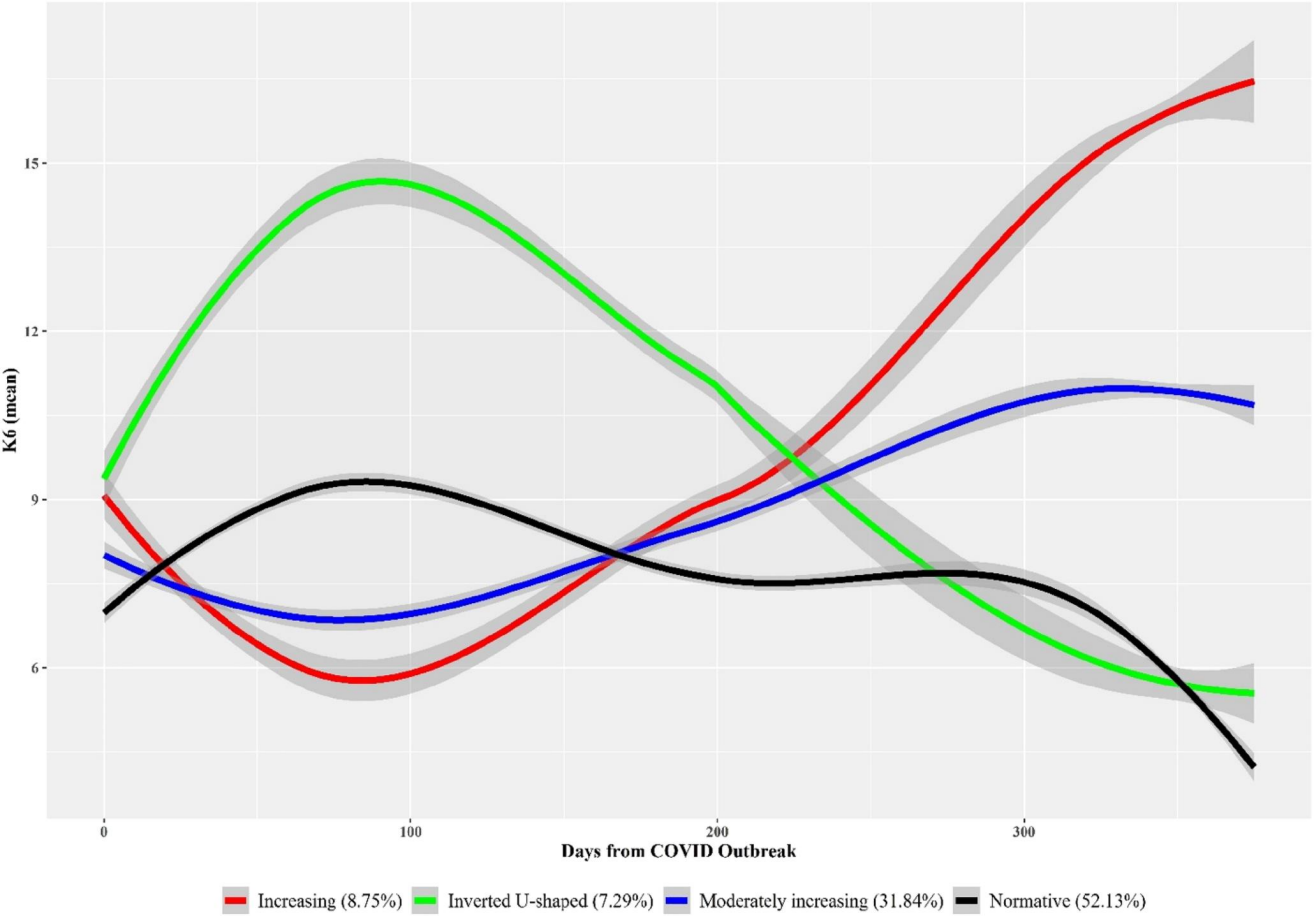
Psychopathology distress trajectories during the first coronavirus disease 2019 (COVID‐19) pandemic year. Trajectories were estimated on the Kessler K6 Scale (K6) scores across the three COVID‐19 survey occasions. Shaded area represents the 95% confidence interval of mean.

Psychological distress trajectory analysis revealed a better fit for the 4‐class model depicting a linear effect of time (SABIC = 186,115.48, AIC = 186,072.05; mean of posterior probabilities for each class = 0.70–0.90). The Table S1 displays the fit indexes derived from all the estimated GMM models (see Supplementary materials). The heterogeneous trajectories of psychological distress across the COVID‐19 measurement occasions are displayed in Figure 1. The first identified class (*increasing class*; 8.75% of participants) was characterized by an increasing distress trend (b coming from a peak of minimal distress over then first assessment wave, on May 2020) following the first COVID‐19 survey (intercept, *B* = −1.32, *Z* = −20.12, *p* < 0.01; time effect, *B* = 0.02, *Z* = 91.61, *p* < 0.01). The second identified class (*inverted U‐shaped class*) comprised 7.29% of participants and was characterized by an elevation in distress (reaching in maximum level over 90 days after the pandemic declaration) during the first wave of the pandemic, followed by a decreasing course of distress thereafter (intercept in comparison with the first class intercept, *B* = 7.46, *Z* = 25.66, *p* < 0.01; time effect, *B* = −0.02, *Z* = −74.06, *p* < 0.01). The third class (so‐called *moderately increasing class*; 31.84% of sample) was characterized by a smooth rise in distress over time after the first COVID‐19 survey (intercept in comparison with the first class intercept, *B* = 1.32, *Z* = 5.37, *p* < 0.01; time effect, *B* = 0.01, *Z* = 54.03, *p* < 0.01). Finally, the fourth class (*normative class*; 52.13% of participants) showed a slight increase in distress during the first COVID‐19 survey (on May 2020 approximately) and a decreasing trend onwards (intercept in comparison with the first class intercept, *B* = 2.70, *Z* = 11.84, *p* < 0.01; time effect, *B* = −0.01, *Z* = −34.45, *p* < 0.01).

### Multinomial logistic regression and multilevel linear regression

Regarding multinomial logistic regression and multilevel linear regression analysis to characterize trajectory class membership, sample size was different across analyses due to the amount of missing data for each outcome. A sample of 1355 subjects was used for these analyses. Attrition statistics for each regression analysis are displayed in Table S2 (see the Supplementary analysis). Significant differences were found across several factors, but they were not meaningful (i.e., with at least medium effect size: Cohen's *d* ≥ 0.50, Cramer's *V* ≥ 0.30, *η* ^2^ ≥ 0.06), except for sex. In this case, a higher number of men had missing data, in comparison to women. For that reason, a smaller number of men were included in the regression analyses.

#### Associations between distress trajectory membership and sociodemographic pre‐COVID‐19 and COVID‐19‐related factors

Multinomial logistic regression was used to study the relationship between the cross‐sectional predictors and psychological distress class membership. The model with covariates (AIC = 3033.03) fitted significantly better to data than the unconstrained model (AIC = 3168.71). Thus, the explanatory power of the model increases with the inclusion of covariates. Covariate coefficients are displayed in Table 2. The *inverted U‐shaped class* membership (in comparison to the *normative class* membership) was associated with higher adolescent psychopathology measured with the SDQ (RRR = 1.08, *p* < 0.01) and lower risk of self‐harm at age 17 (RRR = 0.50, *p* < 0.05), as well as higher risk of financial difficulties during the pandemic lockdown (RRR = 1.62, *p* < 0.05). The *minimally increasing class* membership was associated with higher risk of family conflict during the first year of the pandemic (RRR = 1.44, *p* < 0.05), in comparison to *normative class* membership. Finally, no cross‐sectional predictors were associated with *increasing trajectory class* membership (in comparison to *normative class*).

**TABLE 2:** Multinomial logistic regression to explain psychopathology trajectory class membership.

	Inverted U‐shaped			Minimally increasing			Increasing		
	RRR	CI_95_	Z	RRR	CI_95_	Z	RRR	CI_95_	Z
(Intercept)	0.07	(0.02, 0.25)	−4.01**	0.38	(0.18, 0.79)	−2.57*	0.18	(0.06, 0.55)	−2.98**
Sex (ref.: Male)	0.75	(0.45, 1.22)	−1.16	1.07	(0.82, 1.40)	0.49	0.94	(0.62, 1.42)	−0.31
Ethnicity (ref.: White Caucasian)	0.78	(0.36, 1.69)	−0.63	1.24	(0.87, 1.78)	1.2	1.06	(0.60, 1.89)	0.2
Pre‐COVID (mid‐adolescence) psychopathology									
SDQ (total difficulties)	1.08	(1.03, 1.13)	3.17**	1.02	(0.99, 1.04)	1.2	1.00	(0.96, 1.04)	0
SDQ (prosocial scale)	0.98	(0.86, 1.13)	−0.22	1.02	(0.94, 1.10)	0.39	0.99	(0.88, 1.12)	−0.11
Self‐harm behavior	0.50	(0.28, 0.92)	−2.22*	0.88	(0.65, 1.20)	−0.8	1.14	(0.72, 1.82)	0.56
COVID‐related factors									
Being infected by COVID‐19 (ref.: no)	0.80	(0.37, 1.73)	−0.56	0.84	(0.56, 1.25)	−0.87	0.79	(0.42, 1.51)	−0.71
Family conflict (ref.: no more conflict)									
More conflict during the pandemic	1.17	(0.66, 2.08)	0.54	1.44	(1.07, 1.94)	2.37*	1.12	(0.69, 1.83)	0.47
Financial management (ref.: About the same or better off in comparison to the pre‐pandemic)									
Worse off management during the pandemic	1.62	(1.00, 2.61)	1.97*	1.16	(0.88, 1.53)	1.08	1.24	(0.81, 1.88)	0.99

*Note*: The normative distress trajectory membership was the reference category.

Abbreviations: CI_95_, 95% confidence interval of the RR; RRR, Relative ratio; SDQ, Strengths and Difficulties Questionnaire; Z, Wald's z‐based statistic to test whether loading is significantly different from one.

*p < 0.05; **p < 0.01.

#### Relationship between distress trajectory class membership and the course of loneliness and social support during the first year of the pandemic

In terms of the associations between the distress trajectory class membership and time‐variant social processes (i.e., loneliness and social support), the multilevel linear regression model with covariates, quadratic time term and time*class interaction effects fitted better to data (see Table 3). A sample of 1033 and 1030 participants was used to study the relationship between distress trajectory class membership and the course of loneliness and social support during the first year of the pandemic, respectively.

**TABLE 3:** Multilevel linear regression for loneliness and social support course.

	Loneliness course			Social support course		
	B	SE	t	B	SE	t
(Intercept)	5.02	0.06	93.91**	6.83	0.03	266.71**
Time effect^a^						
Linear	2.91	1.74	1.68	0.24	1.18	0.21
Quadratic	1.72	1.74	0.99	−0.1	1.1	−0.09
Distress trajectory class (ref.: normative)						
Inverted U‐shaped	−0.01	0.14	−0.1	−0.01	0.07	−0.11
Minimally increasing	−0.05	0.08	−0.71	0.01	0.03	0.31
Increasing	0.15	0.12	1.27	0.01	0.06	0.06
Pre‐COVID (mid‐adolescence) psychopathology						
SDQ (total difficulties)	0.58	0.03	16.48**	−0.09	0.02	−5.54**
SDQ (prosocial)	−0.05	0.03	−1.59	0.05	0.02	3.07**
COVID‐related factors						
Being infected by COVID‐19 (ref.: no)	−0.15	0.11	−1.44	0.04	0.05	0,74
Family conflict (ref.: no more conflict)						
More conflict	0.29	0.08	3.37**	−0.04	0.03	−1.11
Financial management (ref.: Same or better off)						
Worse	0.23	0.07	3.04**	−0.09	0.03	−2.61**
Interaction effects^b^						
Linear time effect*Inverted U‐shaped class	−0.06	5.25	−0.01	−4.35	3.85	−1.13
Quadratic time effect*Inverted U‐shaped class	2.56	5.25	0.49	1.75	3.68	0.47
Linear time effect*Minimally increasing class	4.25	2.86	1.48	1.31	1.89	0.69
Quadratic time effect*Minimally increasing class	−1.2	2.86	−0.42	0.65	1.78	0.37
Linear time effect*Increasing class	11.5	4.49	2.56*	−1.59	3.08	−0.51
Quadratic time effect*Increasing class	5.91	4.49	1.31	−0.1	2.9	−0.03
Random‐effects SD	1.04			0.31		
AIC						
Unconstrained	16,231.69			4246.53		
Linear model	15,941.12			4246.92		
Quadratic	15,930.19			4239.48		
Model with interaction term (linear)	15,949.55			4262.65		
Model with interaction term (quadratic)	15,904.39			4225.69		

^a^ Time effects accounts for the outcomes across the three COVID‐19 waves.

^b^ Effects derived from the interaction between the time (wave) effects and the distress trajectory class (in comparison to the reference category = normative trajectory class).

Abbreviations: AIC, Akaike information criterion; B, Loading coefficient; SD, Standard deviation; SE, Standard error; SDQ, Strengths and Difficulties Questionnaire.

*p < 0.05; **p < 0.01.

**FIGURE 2:**
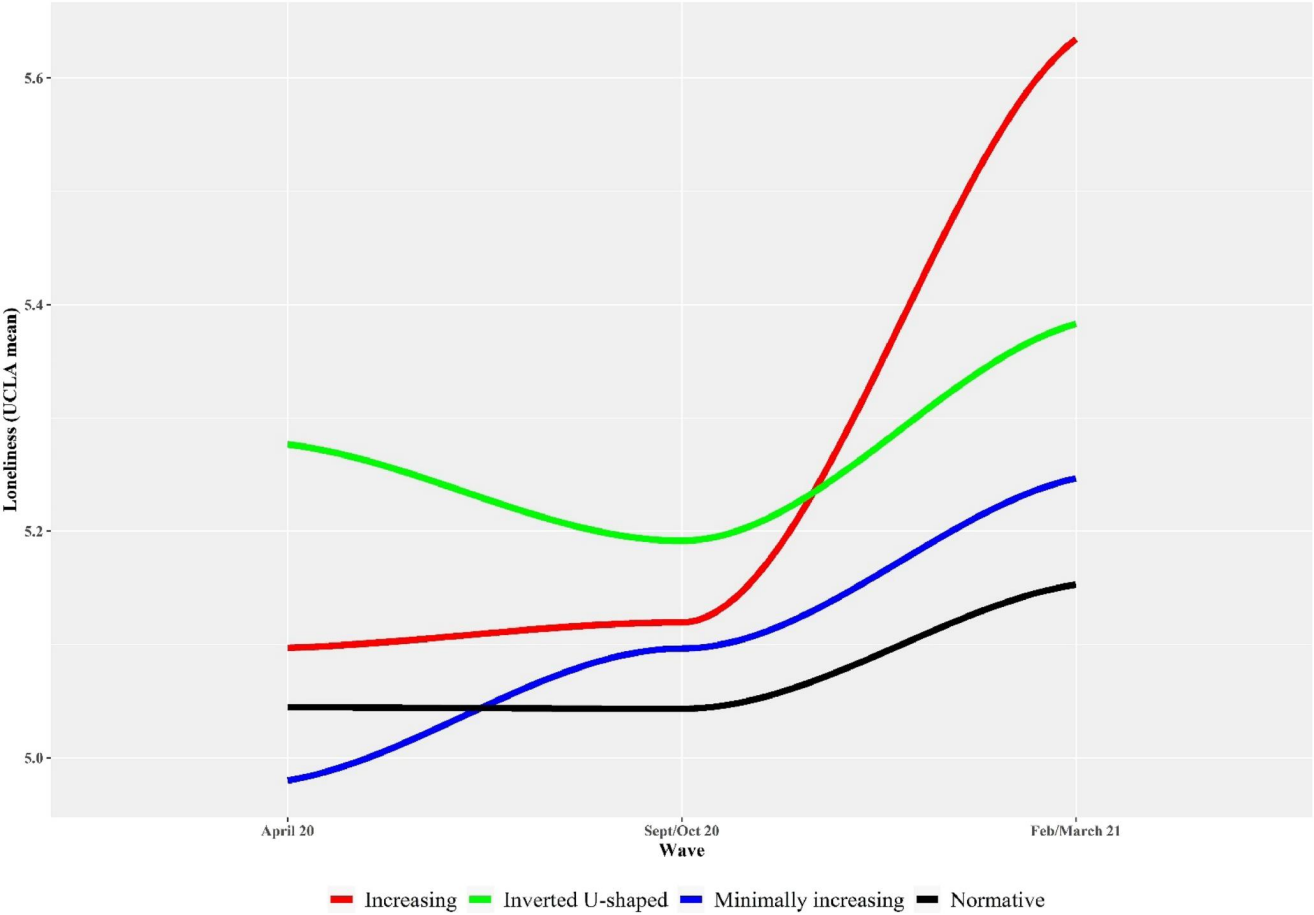
Course of loneliness according to psychopathology distress trajectory class.

Several factors were significantly associated with both the loneliness course (i.e., greater loneliness was related to mental health difficulties at age 17, more family conflict and financial difficulties during the first COVID‐19 years) and social support course (i.e., less social support was related to mental health difficulties at age 17 and poorer prosocial behavior, and financial difficulties during the first COVID‐19 years), but no time main effects were observed for both processes. However, an interaction effect was found between the linear time effect of perceived loneliness and the *increasing class* membership (*B* = 11.5, *p* < 0.05). Figure 2 displays the course of loneliness according to distress trajectory class membership. Participants from the increasing distress trajectory class showed a sharp increase in loneliness starting at the second COVID‐19 wave, in comparison to the rest of participants. No other class membership effect or interaction effects was found for the loneliness and social support models.

## DISCUSSION

Using a large longitudinal cohort of late adolescents, the UK Millennium Cohort Study, this study examined heterogeneity in the changes in psychological distress at 3 time points during the first year of the COVID‐19 pandemic (May 2020, September/October 2020 and February/March 2021). Four distinct trajectories of psychological distress were identified. Furthermore, although perceived social support was stable throughout the assessment period, there was an increase in loneliness during the first year of the pandemic. Larger longitudinal increases in loneliness during the pandemic period were noted among individuals belonging to the elevated distress class, compared to those who experienced less psychological distress. Pre‐pandemic psychopathology was higher among participants who exhibited elevated distress during the first wave of the pandemic. These findings highlight that although the normative group exhibited low and even decreasing distress, a subset of adolescents experienced prolonged and increasing distress in the context of prolonged pandemic‐related PHSM.

Results indicated that there was an overall slight increase in psychological distress across the first year of the COVID‐19 pandemic among UK late adolescents. However, there was significant heterogeneity in the patterns of change over time. Growth mixture modeling identified four latent trajectory classes. On one hand, we observed that half of the participants experienced low and decreasing levels of psychological distress during the pandemic, highlighting the resilience of the majority of late adolescents during the pandemic. This result is consistent with findings from other cohorts (Foster et al., 2023; Manchia et al., 2022). This adaptation could reflect the decreased COVID‐19 related uncertainty over time (Killgore et al., 2020), the progressive adaptation to the crisis situation and the development of alternative socialization strategies in the face of restrictions for in‐person social interactions (Manchia et al., 2022). On the other hand, about 31.1% experienced a small, but significant increase in distress over time and about 8.75% exhibited a larger increase in distress after the first wave of the pandemic. Another 7.29% of participants experienced elevated psychological distress during the first wave of the pandemic, followed by a decrease in distress in subsequent waves of the pandemic. This heterogeneity is in line with other studies who identified distinct psychological distress trajectories during the COVID‐19 pandemic (Pierce et al., 2021; Saunders et al., 2021).

The transition to adulthood is characterized by developmental changes in different life domains and roles, including education, employment, social network, marriage, cohabitation, and parenthood (Scales et al., 2016). Pandemic‐related restrictions led to constrained opportunities in several of these key life domains, highlighting the unique developmental challenges experienced by late adolescents during the COVID‐19 pandemic. Specifically, confinement‐related and physical distancing measures may have constrained opportunities for expansion of social, romantic, and sexual relationships. In the present study, there was a significant increase in loneliness over time. This is consistent with other studies indicating that late adolescents and young adults experienced largest increases in loneliness compared to other age groups (Varga et al., 2021). In contrast, but consistent with other work (Hamza et al., 2021; Laham et al., 2021), there was no change in perceived social support in the present study. This may be because one's perception of the availability of social support may be less dependent on in‐person social interactions, but more strongly related to core attachment‐related beliefs about the availability and responsiveness of close others (Green et al., 2011).

Adolescents experiencing larger increase in psychological distress over time also reported larger increases in loneliness during the pandemic period. This is in line with other studies that observed that loneliness was one of the most robust predictors of psychological distress during the COVID‐19 pandemic (Laham et al., 2021). Although increases in psychological distress and loneliness co‐occurred in the present study, cross‐lagged analysis in a prior longitudinal study indicated that loneliness predicted future depressive symptoms, but not vice‐versa (Cacioppo et al., 2010; Erzen & Çikrikci, 2018; Kraav et al., 2021; Martín‐María et al., 2021). Depression often also leads to a decrease in social relations linked to anhedonia (Brohan et al., 2011; Fernández‐Theoduloz et al., 2019; Hauenstein; 2003; Hopko et al., 2008; Lynch et al., 2021) in addition to the acquisition of a more sedentary lifestyle habits that result in a decrease in social interaction, leading to vicious circle between depression and loneliness (De Moor et al., 2006; Vancampfort et al., 2015).

Conceptual models of loneliness suggest that prolonged loneliness may foster biased social cognition associated with hypervigilance to social threat (Cacioppo & Hawkley, 2009). This biased social information processing may in turn result in more negative social experiences that increase interpersonal stress and risk for psychological distress (Spithoven et al., 2017). In line with this model, prior psychopathology (i.e. elevated SDQ score at age 17) and the presence of family conflict were associated with higher loneliness in the present study. Furthermore, in line with other work (Loibl et al., 2021; Refaeli & Achdut, 2021), greater financial stress was also related to higher loneliness during the COVID‐19 pandemic. Elevated internalizing and externalizing symptoms assessed using the SDQ Difficulties score at age 17 was associated with the inverted U‐shaped trajectory, compared to the other trajectory groups. Individuals in this group experienced the highest levels of psychological distress during the first wave of the pandemic. However, they experienced a gradual decrease in psychological distress during the latter part of the year, suggesting a habituation process, whereby individuals become less reactive or impacted by pandemic‐related stressors over time. Habituation is defined as a decrease in response to repeated stimulation (Thompson, 2010). In prior work, the presence of elevated psychological distress prior to the pandemic moderated changes in distress over time. Although individuals with prior anxiety and depression had overall higher levels of distress, they experienced a smaller increase, and even a decrease in distress in some studies, during the pandemic (Bouter et al., 2022; Hamza et al., 2021; Watkins‐Martin et al., 2021). The PHSMs may have constrained opportunities for in‐person social interactions. However, they may also have reduced exposure to a number of social stressors. This context may have promoted less psychological distress among those who typically display emotion regulation difficulties in response to social stressors. Intriguingly, the presence of a history of non‐suicidal self‐harm was associated with a lower probability of belonging to the inverted U‐shaped trajectory. The negative urgency associated with self‐injury behavior may be associated with distinct emotion regulation strategies (e.g., increased use of alcohol and tobacco) during the pandemic (Essau & de la Torre‐Luque, 2021; Hamza et al., 2015; King et al., 2018). Prior work indicate that higher loneliness during the COVID‐19 pandemic was associated with a decrease in self‐harm among those with high pre‐pandemic self‐harm, suggesting a distinct distress trajectory among this group (Schwartz‐Mette et al., 2022).

For the minimally increasing distress, the presence of family conflict was the only predictor of membership to this trajectory. Some studies have reported increases in family conflict during lockdown periods (Morgül, Kallitsoglou, Essau, & Castro‐Kemp, 2022; Orgilés et al., 2020; Stassart et al., 2021). In a longitudinal study with adolescents, family conflict was associated with larger increases in distress over time (Magson et al., 2021). During the COVID‐19 pandemic, adolescents had less opportunities to interact in person with friends, classmates or colleagues, but spent more time with other household members. The presence of social conflict in the proximal social environment may thus have impeded adjustment to pandemic‐related stressors.

A key strength of the present study is the longitudinal study design with repeated measurements before and during the pandemic, allowing us to examine the influence of developmental antecedents as well as changing social processes during the pandemic. Moreover, a robust analytical strategy was followed. Limitations include the use of non‐validated measures to assess social support and family conflict. In addition, although previous studies have taken into account this type of intentional acts of harming oneself using as a measure the performance of self‐harming behaviors (Hartas, 2023; Uh et al., 2021) this is a non‐validated measure of self‐harm. Similarly, the Kessler Psychological Distress Scale (K6) is a brief scale that does not allow for distinctions between anxiety and depressive symptoms. Furthermore, although the sample was representative at the study outset, differential attrition may have reduced its representativeness. Furthermore, although many participants completed at least one assessment during the COVID‐19 pandemic, only a smaller percentage completed all 3 pandemic assessments. Moreover, late adolescence is a developmental period associated with the onset and a high incidence of anxiety and depressive disorders (Solmi et al., 2022). As such, it is difficult to disentangle the contribution of developmental changes, pandemic‐related stress, or other cohort effects to the change in psychological distress over time.

## CONCLUSION

In conclusion, about 8.75% of late adolescents exhibited a pattern of increasing distress over time, which was associated with concomitant increases in loneliness. Whether adolescents within the increasing distress trajectory will experience a decrease in symptoms once pandemic‐related stressors abate is unclear. Longitudinal follow‐ups will be required to characterize the longer‐term trajectories of psychological distress among these individuals in the post‐pandemic period.

On the other hand, it is also important to note that about 51.2% of late adolescents experienced low levels of psychological distress throughout the first year of the COVID‐19 pandemic. The fact that the majority were resilient in the face of the various pandemic stressors is important. The risk for future pandemics is high (Marani et al., 2021). Furthermore, climate change leading to more frequent extreme weather events may prompt the use of confinement measures restricting in‐person interactions in the future (Longman et al., 2023). Exploring the different trajectories of distress in times of crisis can help confront possible future threats and identify vulnerable populations. In addition, knowing the factors associated with psychological distress will allow us to develop interventions protocols for action in different contexts (e.g., educational, health and family), and to propose specific interventions to target risk factors such as loneliness (Cooper et al., 2021; Loades et al., 2020).

## AUTHOR CONTRIBUTIONS

**Jean‐Philippe Gouin:** Conceptualization, Data curation, Formal analysis, Writing – original draft, Writing – review & editing. **Alejandro de la Torre‐Luque:** Conceptualization, Data curation, Formal analysis, Writing – original draft, Writing – review & editing. **Yolanda Sanchez‐Carro:** Writing – review & editing. **Marie‐Claude Geoffroy:** Conceptualization, Writing – review & editing. **Cecilia Essau:** Conceptualization, Writing – review & editing.

## CONFLICT OF INTEREST STATEMENT

The authors have declared that they have no competing or potential conflicts of interest.

## Ethical considerations

For this study, we used MCS data, registered with the UK data service upon an End User Licence agreement. Existing data use does not require independent ethics committee application. MCS survey participants provided a written consent to participate. Ethical approval for the MCS was obtained from the relevant committees and from the University College London Institute of Education Research Ethics Committee (REC1334).

## Supporting information

Supplementary Material

## Data Availability

Data from the MCS are public and can be accessed online.
