# Supplementary material for: Heterogeneity in the trajectories of psychological distress among late adolescents during the COVID‐19 pandemic
Source: JCPP Adv. 2023 Aug 24;3(4):e12195. doi: 10.1002/jcv2.12195 (PMC10694544; doi:10.1002/jcv2.12195)
Supplement: Supplementary file 1 — Supplementary Material [file JCV2-3-e12195-s001.docx]

**Supporting Information**

**Table S1. Growth mixture model solutions and fit indexes.**

| Class | Time effect | LL | SABIC | AIC | %n in class |
| --- | --- | --- | --- | --- | --- |
| 1 | Linear | -99837.39 | 199696.15 | 199682.79 | 100 |
| 1 | Quadratic | No convergence | |  |  |
| 2 | Linear | -95421.23 | 190879.85 | 190856.46 | 34.51-65.49 |
| 2 | Quadratic | -95312.30 | 190672.66 | 190642.59 | 33.81-66.19 |
| 3 | Linear | -93737.10 | 187527.62 | 187494.21 | 13.44-63.47 |
| 3 | Quadratic | No convergence | |  |  |
| 4 | Linear | -93023.03 | 186115.48 | 186072.05 | 7.29-52.13 |
| 4 | Quadratic | No convergence | |  |  |
| 5 | Linear | -92807.26 | 185699.97 | 185646.52 | 2.82-44.12 |
| 5 | Quadratic | No convergence | |  |  |
| 6 | Linear | -92581.70 | 185264.88 | 185201.40 | 2.44-47.51 |
| 6 | Quadratic | No convergence | |  |  |

**Note.** ‘Class’ refers to number of classes considered in each model. ‘%n in class’ refers to range of percentage of participants in each class. The days from the WHO’s statement of pandemic was used as a time factor. Quadratic models included both the linear and quadratic omponent.

LL = Maximum log-likelihood estimator for model convergence. SABIC = Sample-adjusted Bayesian information criterion. AIC = Akaike information criterion.

**Table S2. Sample features and attrition analysis for the regression models.**

|  | **Multinomial regression** | |  |  | **Loneliness course regression** | | |  | **Social provisions course regression** | | |  |
| --- | --- | --- | --- | --- | --- | --- | --- | --- | --- | --- | --- | --- |
|  | **Dropped-out sample** | **Sample in analysis** | **Contrast test** | **ES** | **Dropped-out sample** | **Sample in analysis** | **Contrast test** | **ES** | **Dropped-out sample** | **Sample in analysis** | **Contrast**  **test** | **ES** |
| N | 3659 | 1355 |  |  | 3981 | 1033 |  |  | 3984 | 1030 |  |  |
| Sex (%male) | 44.22 | 26.42 | 130.45 | 0.16 | 42.85 | 26.14 | 95.28** | 0.14 | 42.87 | 26.02 | 96.63** | 0.14 |
| Age (in years) | 19.26 (0.45) | 19.3 (0.47) | -1.34 | 0.09 | 19.27 (0.46) | 19.29 (0.46) | -0.87 | 0.06 | 19.27 (0.46) | 19.29 (0.46) | -0.81 | 0.06 |
| country  (%England resident) | 65.31 | 66.35 | 3.27 | 0.03 | 65.41 | 66.31 | 1.91 | 0.02 | 65.49 | 66.02 | 1.86 | 0.02 |
| **Pre-COVID psychopathology** | | | | | | | | | | | | |
| Total Difficulties^1^ | 11.47 (5.63) | 10.9 (5.36) | 3.27** | -0.1 | 11.46 (5.6) | 10.76 (5.37) | 3.66** | -0.13 | 11.46 (5.6) | 10.77 (5.37) | 3.56** | -0.12 |
| Prosocial behavior^1^ | 7.96 (1.71) | 8.26 (1.56) | -5.78** | 0.18 | 7.98 (1.7) | 8.28 (1.54) | -5.56** | 0.18 | 7.98 (1.7) | 8.28 (1.55) | -5.5** | 0.18 |
| Self-harm (%yes) | 24.55 | 28.27 | 6.82** | 0.04 | 24.9 | 28.17 | 4.36* | 0.03 | 24.82 | 28.45 | 5.36* | 0.03 |
| **COVID-19 factors** |  |  |  |  |  |  |  |  |  |  |  |  |
| Being infected (%yes) | 13.48 | 11.81 | 2.11 | 0.02 | 13.11 | 12.39 | 0.29 | 0.01 | 13.13 | 12.33 | 0.37 | 0.01 |
| Family conflict  (%more than the pre-pandemic) | 27.46 | 30.85 | 2.98 | 0.03 | 28.89 | 30.11 | 0.35 | 0.01 | 28.9 | 30.1 | 0.34 | 0.01 |
| Financial management (%worse than the pre-pandemic) | 30.83 | 24.21 | 12.2** | 0.05 | 29.56 | 23.72 | 9.63** | 0.04 | 29.49 | 23.79 | 9.17** | 0.04 |
| Distress psychopathology  class | | | 4.33 | 0.03 |  |  | 2.16 | 0.02 |  |  | 1.46 | 0.02 |
| Normative | 53.95 | 51.12 |  |  | 52.85 | 54.33 |  |  |  |  |  |  |
| Increasing | 9.06 | 10.17 |  |  | 9.38 | 9.33 |  |  |  |  |  |  |
| Inverted U-shaped | 7.13 | 4.96 |  |  | 7.01 | 4.67 |  |  |  |  |  |  |
| Moderately increasing | 29.87 | 33.75 |  |  | 30.76 | 31.67 |  |  |  |  |  |  |
| Psychopathology score^2^ |  |  | 5.88* | 0 |  |  | 5.58* | 0 |  |  | 6.28* | 0 |
| COVID-19 wave 1 | 8.73 (5.3) | 8.17 (4.97) |  |  | 8.61 (5.25) | 8.16 (4.95) |  |  | 8.59 (5.24) | 8.18 (4.97) |  |  |
| COVID-19 wave 2 | 8.56 (5.53) | 8.23 (5.09) |  |  | 8.58 (5.53) | 8.2 (5.08) |  |  | 8.57 (5.53) | 8.21 (5.08) |  |  |
| COVID-19 wave 3 | 8.37 (5.69) | 9.15 (5.31) |  |  | 8.49 (5.68) | 9.01 (5.23) |  |  | 8.5 (5.69) | 8.99 (5.22) |  |  |
| Loneliness^3^ |  |  | 5.38* | 0 |  |  | 5.38* | 0 |  |  | 6.44* | 0 |
| COVID-19 wave 1 | 5.2 (1.75) | 5.13 (1.67) |  |  | 5.16 (1.72) | 5.17 (1.67) |  |  | 5.16 (1.72) | 5.16 (1.68) |  |  |
| COVID-19 wave 2 | 5.24 (1.81) | 5.14 (1.69) |  |  | 5.24 (1.81) | 5.15 (1.7) |  |  | 5.24 (1.81) | 5.14 (1.7) |  |  |
| COVID-19 wave 3 | 5.21 (1.83) | 5.43 (1.74) |  |  | 5.24 (1.82) | 5.41 (1.75) |  |  | 5.24 (1.82) | 5.4 (1.75) |  |  |
| Social support^4^ | |  | 3.42 | 0 |  |  | 3.46 | 0 |  |  | 3.42 | 0 |
| COVID-19 wave 1 | 6.75 (0.67) | 6.82 (0.63) |  |  | 6.76 (0.67) | 6.82 (0.62) |  |  | 6.75 (0.67) | 6.83 (0.62) |  |  |
| COVID-19 wave 2 | 6.79 (0.7) | 6.8 (0.64) |  |  | 6.79 (0.7) | 6.81 (0.63) |  |  | 6.78 (0.71) | 6.81 (0.62) |  |  |
| COVID-19 wave 3 | 6.79 (0.7) | 6.8 (0.69) |  |  | 6.78 (0.71) | 6.82 (0.67) |  |  | 6.78 (0.71) | 6.82 (0.68) |  |  |

**Note.** Percentage of cases are displayed for dichotomous and categorical variables. Mean and standard deviation (between brackets) are displayed for continuous variables. A different sample was used for each of the prediction analyses (multinomial regression, loneliness course regression, social provisions course regression).

The pre-COVID data were collected in 2018, when cohort members were 17 years old. The COVID-19 data were collected across three waves: COVID-19 wave 1 (May 2020), COVID-19 wave 2 (September/October 2020) and COVID-19 wave 3 (February/March 2021).

The t -based tests (continuous variables) and χ^2^ tests (dichotomous/categorical variables) were used as contrast test statistics. For the time-variant factors (psychopathology score, loneliness and social support) the Snedecor’s F statistic from the repeated-measure analysis of the variance was used. Effect size (ES) estimates were the Cohen’s d for continuous variables, the η^2^ for the time-variant factors, and Cramer’s V for non-continuous ones.

^1^ Derived from the Strength and Difficulties Questionnaire (SDQ).

^2^ Coming from the Kessler K6 Scale (K6).

^3^ Derived from the 3-item UCLA Loneliness Scale.

^4^ Derived from the 3-item Social Provisions Scale.

* p < .05; ** p < .01.
